# Esophageal deviation using the transesophageal echocardiography probe during atrial fibrillation radiofrequency ablation: a prospective observational study

**DOI:** 10.3389/fcvm.2026.1917536

**Published:** 2026-09-10

**Authors:** Fabien Squara, Sok-Sithikun Bun, Camille Verdan, Didier Scarlatti, Emile Ferrari

**Affiliations:** 1 Service de Cardiologie, Centre Hospitalier Universitaire de Nice, Nice, France; 2 Unité de Recherche Clinique Côte d'Azur (UR2CA), Université Côte d'Azur, Nice, France

**Keywords:** ablation, atrial fibrillation, barium, deviation, esophagus, radiofrequency, transesophageal echocardiography

## Abstract

**Background:**

Esophageal injury is a recognized complication of atrial fibrillation (AF) radiofrequency ablation, during pulmonary vein isolation (PVI) or posterior wall ablation. Mechanical esophageal deviation has emerged as a promising preventive strategy, however, commercially available deviation tools are not accessible outside the United States. We evaluated the feasibility and effectiveness of using the transesophageal echocardiography (TEE) probe—readily available in many electrophysiology labs—for esophageal deviation during AF ablation.

**Methods:**

In this prospective observational study, patients undergoing PVI ± additional atrial ablation for AF were treated using esophageal deviation (Dev-Group) or using standard care (Std-Group) depending on the operating electrophysiologist. In the Dev-Group, esophageal position was identified using barium ingestion, and the TEE probe was manipulated to achieve maximal displacement away from posterior pulmonary vein (PV) ostia. Deviation of esophageal trailing edge >10 mm from PV was defined as successful. Esophageal temperature was monitored in both groups using a multi-sensor probe.

**Results:**

Sixty patients (30 per group; 60 PV pairs each) were included. In the Dev-Group, 78% (47/60) of PV pairs achieved >10 mm esophageal separation vs. 30% (18/60) at baseline (*p* < 0.001); PV-esophagus overlap dropped from 52% (31/60) at baseline to 2% (1/60) after deviation (*p* < 0.001). Temperature rises >38 °C were observed during 2 radiofrequency applications in the Dev-Group versus 75 in the Std-Group (*p* < 0.001). No esophageal injuries were reported.

**Conclusion:**

TEE probe-guided esophageal deviation appears as a practical and effective technique for reducing esophageal proximity and thermal exposure during AF ablation, offering a valuable safety enhancement where dedicated tools are unavailable.

## GRAPHICAL ABSTRACT

**Figure:**
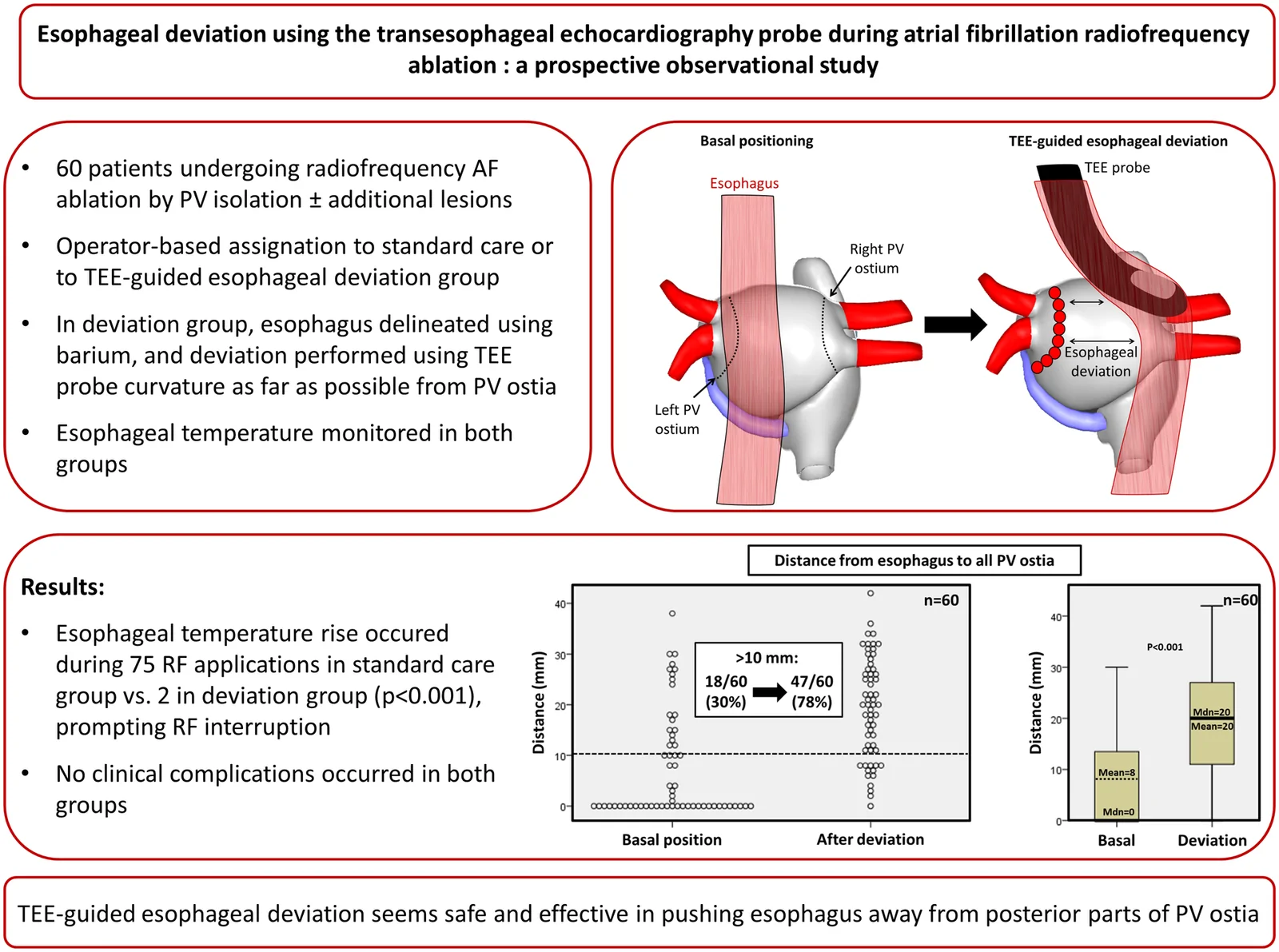

## Introduction

Catheter ablation of atrial fibrillation (AF), particularly pulmonary vein isolation (PVI), has become a mainstay in the management of symptomatic AF (1). Despite increasing use of pulsed-field ablation (PFA), radiofrequency still remains a commonly used energy for AF ablation (2), which exposes adjacent structures to thermal injury. The proximity of the esophagus to the posterior wall of the left atrium makes it vulnerable to unintended heating, with the risk of complications ranging from ulceration to atrioesophageal fistula — a rare but often fatal outcome (3, 4).

Numerous strategies have been investigated to mitigate esophageal injury, including esophageal temperature monitoring, modulation of ablation power and duration, and active esophageal cooling (5). Among emerging mechanical strategies, esophageal deviation — the intentional lateral displacement of the esophagus away from the ablation area — has demonstrated promising results. Initially, esophageal deviation was described in observational studies using non-dedicated devices, such as trans-esophageal echocardiography (TEE) probe (6, 7), gastroenterology endoscope (8), or malleable metal stylet within a plastic tube (9, 10). While these studies sometimes included a large number of patients, they consistently lacked a control group, and esophageal deviation was often performed by another operator, thus requiring additional resources. Nevertheless, these deviation techniques seemed to allow sufficient esophageal displacement for improving radiofrequency application safety in the majority of the patients.

More recently, some commercially available tools have been released, such as the Esosure®, the DV8 Retractor®, and the Esolution® devices. While studies evaluating these systems demonstrated positive results (11–13), such devices induce additional procedural costs, and none of them is available outside the United States.

Given the widespread availability of TEE probes in electrophysiology labs and their quite common use for transseptal punctures guidance in Europe (14), their repurposing for esophageal deviation offers a potentially cost-effective and readily accessible solution. The aim of this study was to further evaluate the feasibility and efficacy of esophageal deviation using TEE in a prospective observational study, when TEE is solely manipulated by the electrophysiologist performing the ablation procedure.

## Methods

### Study design and population

This study was conducted at the University Hospital of Nice, from January 2024 to October 2024. This was a prospective, observational study on patients undergoing radiofrequency AF ablation with PVI ± additional atrial ablation. Patients were divided into two groups based on the operating electrophysiologist, during the same time period: one operator performed esophageal deviation (Dev-Group) using the TEE probe at the time of radiofrequency application on the posterior wall of the left atrium (LA); the other operator used standard care (Std-Group) and patients received conventional ablation without esophageal deviation. In both groups, esophageal temperature was monitored (see further for details). Patients with known esophageal disease or prior esophageal surgery were excluded. Institutional review board approved this research. All patients signed a written informed consent document. This work complies with the declaration of Helsinki.

### Ablation protocol in the standard care group

Patients treated by author S-SB were allocated to Std-Group. Procedures were performed under general anesthesia. Femoral vein accesses were guided by vascular echography. A quadripolar or decapolar catheter was inserted within the coronary sinus. Electroanatomical mapping of the left atrium (LA) was performed with the Carto system V7, using a multielectrode diagnostic catheter (Biosense Webster Inc., Irvine, CA, USA) inserted transseptally via a non-steerable sheath (Fast-Cath SL0; Abbott, Abbott Park, IL, USA). Transseptal punctures were performed under TEE guidance. After successful transseptal access, TEE probe was slightly withdrawn and left in the upper part of the esophagus for the rest of the procedure. A three-sensor esophageal thermal probe (Sensitherm™; Abbott) was inserted within the esophagus for temperature monitoring throughout the ablation procedure.

Point-by-point radiofrequency ablations (35-45W) were performed with irrigated 3.5 mm tip force-sensing catheter (Thermocool SmartTouch SF, Biosense Webster inc.) with endpoint of circumferential pulmonary veins (PV) disconnection, following the CLOSE protocol (15) (≥550 AU anterior, ≥400 posterior, ≤6 mm inter-lesion distance). The choice of performing additional linear ablations outside the PV was left to the operator; when performed, the endpoint was block across the lines. Associated atrial tachyarrhythmia ablation was performed. In cases without AF or atrial tachyarrhythmia termination at the end of the procedure, an electrical cardioversion was performed.

Esophageal temperature was carefully monitored when ablation was performed on the posterior wall (either at the posterior parts of the PVs, or when posterior wall ablation was performed). Position of the thermal probe was adjusted using fluoroscopy for maintaining the closest distance to the ablation catheter. Increase of esophageal temperature was defined as temperature rise >38 °C during RF application, prompting immediate RF interruption. The number of radiofrequency applications inducing temperature increase was recorded for each patient, and the distance between the ablation catheter and the closest sensor of the thermal probe was measured in postero-anterior fluoroscopy incidence.

Following the procedure, a proton-pump inhibitor (pantoprazole 30 mg) was prescribed for 1–2 months duration.

### Ablation protocol in the deviation group

Patients treated by author FS were allocated to Dev-Group. This author was routinely performing esophageal deviation using the TEE probe since >12 months prior to this study. In the Dev-Group, procedural protocol of the Std-Group was followed, but in addition, the esophagus was delineated using barium, deviated, and its distance from the posterior parts of PV ostia was measured.

Specifically, a 12F nasogatric or orogastric tube was inserted to the upper part of the esophagus after general anesthesia induction, before TEE insertion. Procedure then began, and electroanatomical mapping of the LA was performed. Using the ablation catheter, several fluoroscopic landmarks were registered along the posterior part of both PV ostia, where the ablation encirclement line was planned. These fluoroscopic landmarks were then linked to form a line on fluoroscopy, materializing the posterior parts of PV ostia.

Then, baseline course of the esophagus was delineated using postero-anterior fluoroscopy after injection of 25-50cc of a 50% barium solution into the esophagus. The distance between the closest edge of the esophagus and the posterior parts of PV ostia was measured on the fluoroscopy software. When the esophagus overlapped with one PV ostium (posterior part of the PV ostium between the two esophageal edges), the distance was noted to be 0 mm.

Esophageal deviation was then attempted, by carefully manipulating the TEE probe (X7-2 T, Philips Medical, Best, Netherlands). This was performed solely by the operating electrophysiologist, by either using a sterile transparent draping on the TEE handle and body, or by changing gloves after each TEE manipulation. First, the TEE probe was usually gently advanced without curvature to the lower part of the esophagus. Then, the TEE was curved rightwards or leftwards, by adjusting the probe's curvature and rotation using fluoroscopy. The curvature was then fixed on the steering knob, and the TEE probe was gently withdrawn until the maximal deviated esophageal zone reached the level of the posterior PV part. Of note, the TEE probe was never advanced further in the esophagus with a fixed curvature, for avoiding excessive parietal force applied to the esophagus.

Deviation was performed rightwards—as far as possible from left PV ostia—and leftwards—as far as possible from right PV ostia –. After rightwards and leftwards deviation, distances were measured from each esophageal border (leading edge and trailing edge) to the posterior parts of both PV ostia. The most important measure was the distance from the trailing edge of the esophagus to the left PV ostium (during rightwards deviation) and to the right PV ostium (during leftwards deviation), indicating the safety distance between the esophagus and the ablation line. The rate of overlapping esophagus with right or left PV ostium was assessed before and after deviation. Esophageal diameter was measured at mid-PV level at baseline (with the TEE probe being placed in the upper esophagus) and during deviation with the TEE probe, for assessing esophageal stretch induced by displacement.

Deviation of the esophagus was considered successful when the shortest distance between the trailing edge and the PV ostium was > 10 mm. Of note, there is no consensus regarding the most accurate distance cut-off between the ablation spot and the esophagus for ensuring esophageal safety. Some studies evaluated the relation between esophageal temperature and the distance between the ablation catheter and esophageal trailing edge, and reported a distance < 10 mm^12^ or < 20 mm^13^ to be associated with significant temperature increase (>38 to 38.5 °C), but no study correlated this distance to the incidence of esophageal injury. Since esophageal temperature is not an accurate predictor of esophageal injury (11, 16), we rather based our safety distance cut-off on the previous study by Nakagawa et al. (17) demonstrating that RF lesions on beating hearts at moderate, high or very-high power induce maximal lesions depth of 6.7 ± 0.5 mm and maximal lesion diameter of 12.0 ± 0.6 mm (and thus, a lesion radius of 6 mm). Thus, a distance between the ablation spot and the esophageal trailing edge > 10 mm appeared as a clinically pertinent distance for defining successful deviation.

The position of the temperature probe was adjusted during ablation procedure for maintaining the closest distance to the ablation catheter. Specifically in the Dev-Group, the temperature probe was manipulated to ensure that it was positioned closest to the esophageal trailing edge, lateral to the TEE probe.

Following the procedure, a proton-pump inhibitor (pantoprazole 30 mg) was prescribed for 1–2 months duration.

### Follow-up

Patients were discharged the day following ablation procedure, in the absence of complications. Complications were recorded at the time of the hospitalization or during follow-up. Follow-up visits with 24 h Holter ECG recordings were scheduled every 3 months during the first 12 months post-ablation, then at the discretion of the referent cardiologist. Esophageal endoscopy was only discussed in case of clinical suspicion of esophageal injury, namely in case of suspected digestive bleeding, in case of dysphagia, or if sore-throat sensation (quite common after intubation) was persistent after 7 days.

### Statistical analysis

Categorical variables were presented as percentages. Distribution of continuous variables was assessed using Kolmogorov–Smirnov test. Normally distributed continuous variables were presented as mean and standard deviation, and non-normally distributed continuous variables as median and interquartile range. Groups were compared by a parametric *t*-test or non-parametric Mann–Whitney test as appropriate. Categorical variables were compared using Chi-2 test.

Statistical significance was defined by a *P* value < 0.05.

## Results

### Clinical and procedural characteristics

We included 60 patients, 30 in the Dev-Group and 30 in the Std-Group (60 PV pairs each). Demographics and baseline clinical characteristics were similar between groups, as shown in Table 1. Specifically, the prevalence of any cardiopathy, LVEF, left atrial volume, and CHADS-VA score were comparable in both groups. Around half of the patients were treated for persistent AF (53% in Dev-Group, vs. 50% in Std-Group; *p* = 0.796).

**Table 1:** Patients characteristics.

Variable	Deviation group (*n* = 30)	Standard care group (*n* = 30)	*P* value
Age (y)	67.3 ± 12.3	67.3 ± 9.5	0.991
Female gender	12 (40%)	7 (23%)	0.165
High blood pressure	14 (47%)	11 (37%)	0.432
Diabetes mellitus	5 (17%)	6 (20%)	0.739
Dyslipidemia	5 (17%)	8 (27%)	0.347
Cardiopathy (any)	13 (43%)	14 (47%)	0.795
LVEF (%)	60 [45–60]	56 [40–60]	0.295
Stroke	3 (10%)	5 (17%)	0.448
CHADS-VA Score	3 [1–3]	3 [1–3]	0.898
Left atrial volume (mL/m2)	40 ± 9.7	44 ± 16.1	0.308
Direct anticoagulant treatment	30 (100%)	28 (93%)	0.150
Persistent atrial fibrillation	16 (53%)	15 (50%)	0.796

PV isolation was performed in every patient of both groups, and the prevalence of posterior wall ablation (17% for both groups; *p* = 1.000) and roof line ablation (33% in Dev-Group, vs. 30% in Std-Group; *p* = 0.781) were comparable, thus inducing comparable esophageal exposure in the two study groups (Table 2). The only difference in the ablation plan was a more prevalent Marshall vein ethanol infusion+lateral mitral isthmus ablation in the Dev-Group (23% vs. 0%, *p* = 0.005), due to different ablation habits of the two operators for patients presenting with persistent AF and in AF at the time of the procedure.

**Table 2:** Procedural characteristics and complications.

Variable	Deviation group (*n* = 30)	Standard care group (*n* = 30)	*P* value
Left atrial ablation set			
Pulmonary vein isolation	30 (100%)	30 (100%)	NA
Posterior line or wall ablation	5 (17%)	5 (17%)	1.000
Roof line ablation	10 (33%)	9 (30%)	0.781
Lateral mitral isthmus ablation+vein of Marshall ethanol infusion	7 (23%)	0	0.005
Complications			
Clinical esophageal complications	0	0	NA
Groin hematoma requiring surgical revision	0	0	NA
Tamponnade	0	0	NA
Stroke	0	0	NA

### Esophageal data

Study results are summarized in the **Graphical Abstract**.

In the Dev-Group, the total amplitude of the esophageal trailing edge displacement was 29.7 ± 10.5 mm. Specifically, esophageal trailing edge displacement was 15.7 ± 9.6 mm rightwards and 14.1 ± 9.0 mm leftwards, compared to baseline positioning (Table 3). Mean esophageal diameter was 20.2 ± 5.3 mm at baseline, and esophageal diameter stretch during deviation was 3.5 ± 4.2 mm (maximum 13 mm).

**Table 3:** Esophageal data.

Variable	Deviation group (*n* = 30)		Standard care group (*n* = 30)	*P* value
Esophageal data	Baseline positioning	After deviation		
Total amplitude of esophageal trailing edge deviation (mm)	29.7 ± 10.5			
Rightwards deviation of the esophageal trailing edge (mm)	15.7 ± 9.7			
Leftwards deviation of the esophageal trailing edge (mm)	14.1 ± 9.0			
Esophageal diameter stretch during deviation (mm)	3.5 ± 4.2			
Distance between PV ostia and esophagus (mm)	0 [0–13.8]	20 [11–27]		<0.001
Overlap between PV ostium and esophagus	31/60 (52%)	1/60 (2%)		<0.001
>10 mm separation between PV ostium and esophagus	18/60 (30%)	47/60 (78%)		<0.001
Total number of esophageal temperature increase >38 °C	2		75	<0.001
Patients in whom radiofrequency induced esophageal temperature increase >38 °C	2/30 (7%)		27/30 (90%)	<0.001
Number of radiofrequency applications per patient inducing esophageal temperature increase >38 °C	0 [0–0]		3 [2–4]	<0.001
Distance between thermal probe and ablation catheter in cases of temperature increase >38 °C (mm)	4 [0–8]		3 [1–4]	1.000

**Figure 1:**
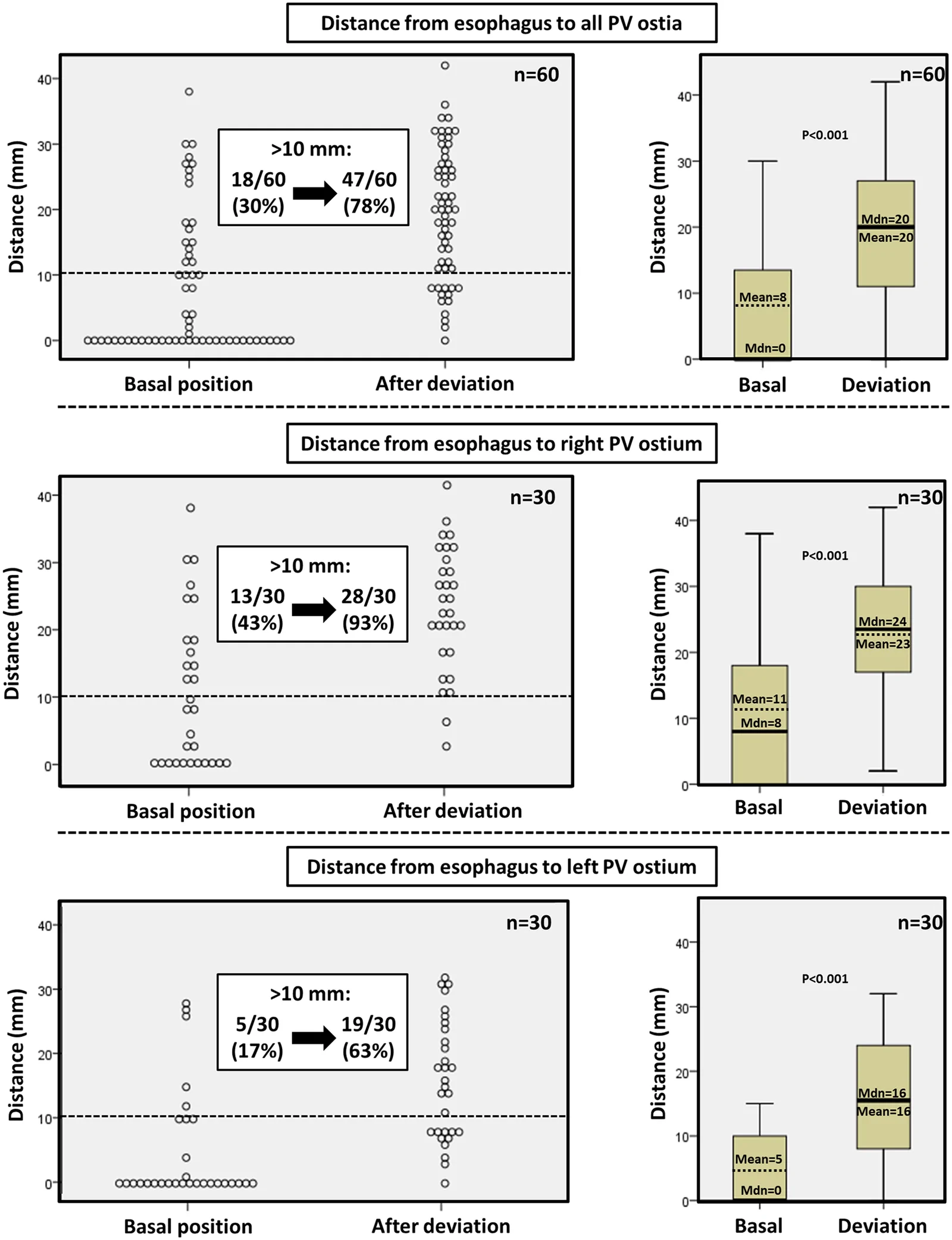
Diagrams depicting basal and post-deviation distances from esophagus to all PV ostia (top), right PV ostia (middle), and left PV ostia (bottom).

**Figure 2:**
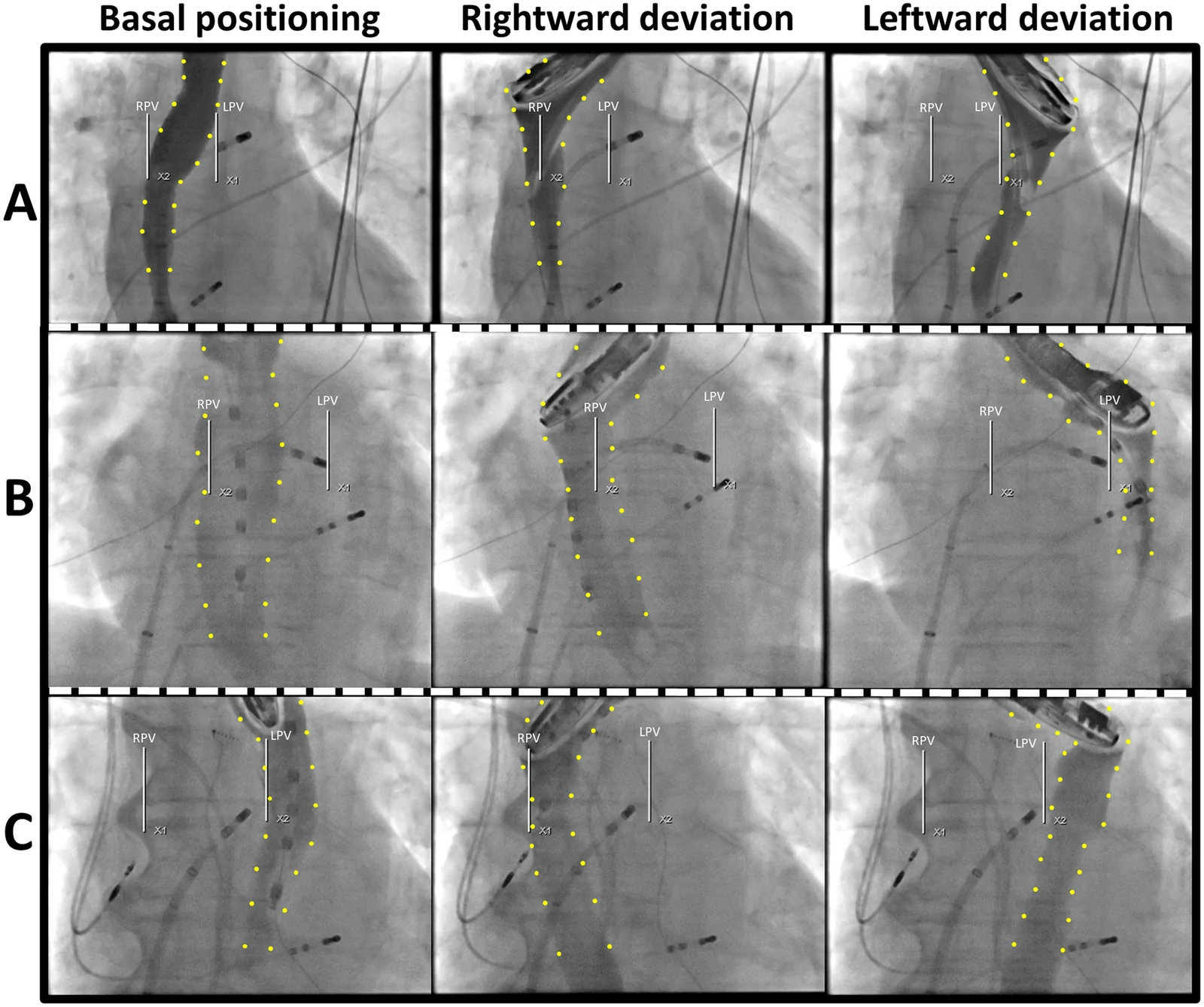
Three examples of successful esophageal deviation, with distance between esophageal trailing edge and both PV ostia > 10 mm after deviation. Posterior parts of each PV ostium are materialized on fluoroscopy by white vertical lines. The course of the esophagus is highlighted with yellow dots for facilitating visualization. **(A)** Esophagus partially overlapping with both PV ostia at baseline. After deviation, esophagus – left PV distance = 15 mm, and esophagus – right PV distance = 26 mm. **(B)** Esophagus overlapping with right PV ostium at baseline. After deviation, esophagus – left PV distance = 32 mm, and esophagus – right PV distance = 34 mm. **(C)** Esophagus overlapping with left PV ostium at baseline. After deviation, esophagus – left PV distance = 24 mm, and esophagus – right PV distance = 42 mm.

**Figure 3:**
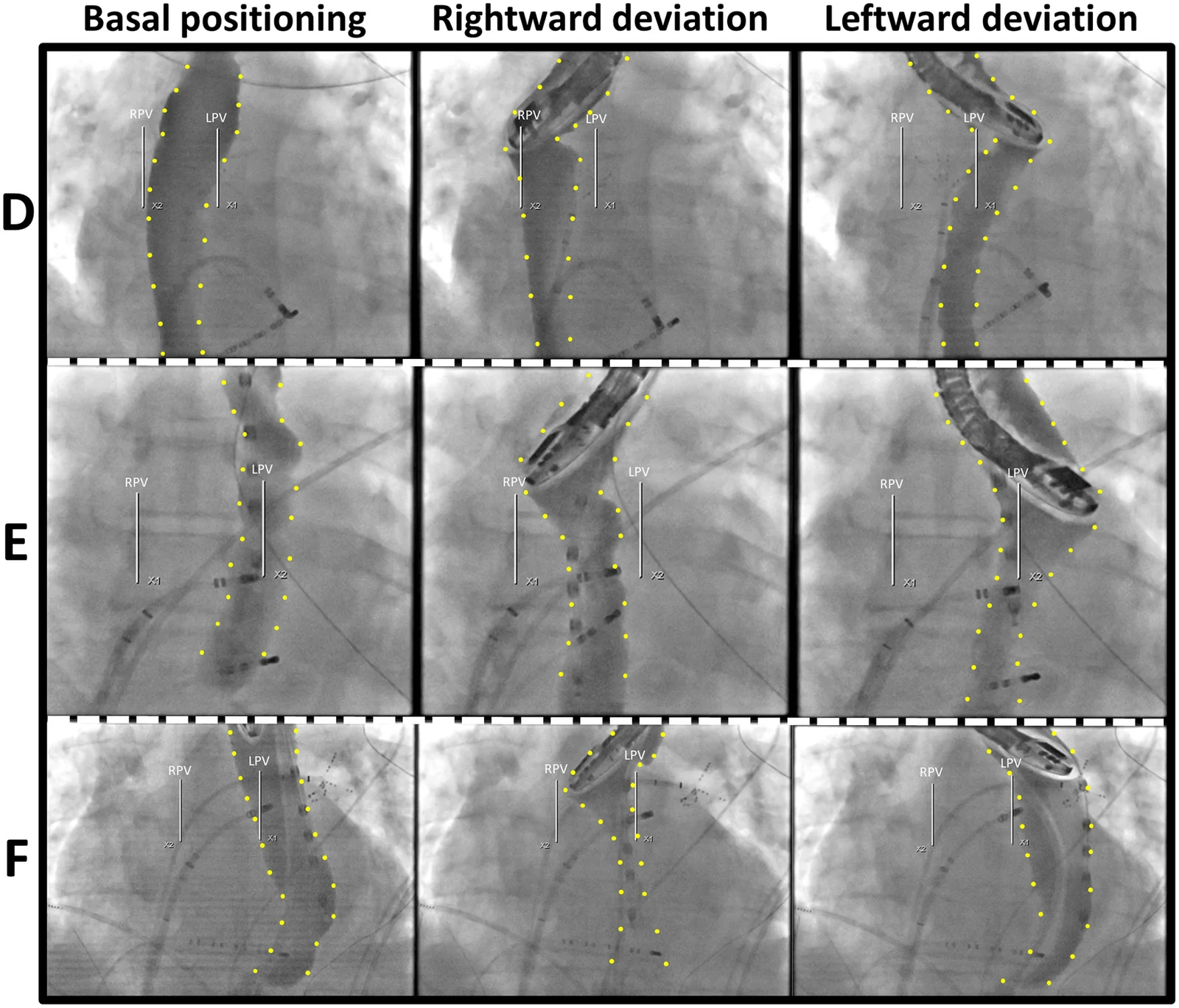
Three examples of partially successful, or unsuccessful esophageal deviation. Posterior parts of each PV ostium are materialized on fluoroscopy by white vertical lines. The course of the esophagus is highlighted with yellow dots for facilitating visualization. **(D)** Esophagus overlapping with left PV ostium and very close (2 mm) to right PV ostium at baseline. After rightward deviation, esophagus – left PV distance is limited to 8 mm. Leftward deviation is successful, with an esophageal – right PV distance of 20 mm. **(E)** Esophagus overlapping with left PV ostium at baseline. Rightward deviation yields a distance from esophagus to left PV of only 6 mm. Leftward deviation is limited, but esophagus – right PV distance is still 32 mm due to leftward basal positioning of the esophagus. **(F)** Esophagus overlapping with left PV ostium at baseline. Deviation is very limited on both sides, with an esophagus still overlapping with left PV ostium (and 34 mm to right PV ostium). Ablation at the posterior left PV ostium induced esophageal temperature increase >38 °C.

Distance from the esophagus to the posterior part of the PV ostia increased from 0 [0-13.8] mm at baseline to 20 [11–27] mm after deviation (*p* < 0.001; Figure 1). Overlap between PV ostium and esophagus dropped from 52% (31/60) at baseline to 2% (1/60) after deviation (*p* < 0.001). Also, 78% (47/60) of PV pairs achieved >10 mm esophageal separation after deviation vs. 30% (18/60) at baseline (*p* < 0.001). More specifically, leftward esophageal deviation allowed >10 mm separation between right PV ostia and esophagus in 93% (28/30) PV pairs vs. 43% (14/30) PV pairs at baseline (*p* < 0.001), and rightwards deviation allowed >10 mm separation between left PV ostia and esophagus in 63% (19/30) PV pairs vs. 17% (5/30) PV pairs at baseline (*p* < 0.001). Figure 2 shows examples of successful esophageal deviation, and Figure 3 shows examples of partially successful or unsuccessful esophageal deviation. Also see Supplementary video for detailed imaging during deviation.

Esophageal temperature rises >38 °C were observed during 2 radiofrequency applications in the Dev-Group versus 75 in the Std-Group (*p* < 0.001). This translated into a number of radiofrequency applications per patient inducing temperature increase >38 °C of 0 [0–0] in the Dev-Group vs. 3 [2–4] in the Std-Group (*p* < 0.001). Only 2/30 (7%) patients of the Dev-Group encountered esophageal temperature rise >38 °C in the Dev-Group vs. 27/30 (90%) in the Std-Group. When esophageal temperature increase occurred, the distance between the closest sensor of the thermal probe and the ablation catheter was similar in the Dev-Group and the Std-Group (4 [0–8] mm vs. 3 [1–4] mm respectively, *p* = 1.000). Including the 77 occurrences of temperature increases in both groups, the longest distance between the thermal probe and the ablation catheter was 12 mm.

### Follow-up

No acute complication was documented after ablation procedure in both groups. Esophageal endoscopy was not performed in any patient because no esophageal complication was clinically suspected. No late esophageal complication occurred during the follow-up of 203 ± 113 days after procedure.

## Discussion

This prospective observational study demonstrates that mechanical esophageal deviation using a standard TEE probe is feasible and effective in reducing esophageal proximity and thermal exposure during AF radiofrequency ablation. Compared with standard care, TEE-guided deviation resulted in a marked increase in the distance between the esophagus and posterior PV ostia, a near-complete abolition of PV–esophagus overlap, and also a dramatic reduction in esophageal temperature rises during ablation on the posterior wall.

### Mechanical efficacy of TEE-guided esophageal deviation

The primary finding of this study is the magnitude of esophageal displacement achievable with a TEE probe. The mean trailing-edge displacement approaching 30 mm, with a median post-deviation esophagus–PV distance of 20 mm, compares favorably with results reported using dedicated commercial deviation devices that don't embed suction system. Namely, the DV8 Retractor® (Manual Surgical Sciences, MN, USA) deviation capabilities have been previously evaluated by Bhardwaj et al. (13), who found a distance of 18.4 ± 8.7 mm between PV ostium and esophagus after deviation, which is strikingly close to our results.

However, systems incorporating active esophageal suction appear to yield particularly favorable results, with a mean post-deviation distance of 33.5 ± 11.3 mm between the PV and the esophagus when using the *ESO*lution® device (S4 Medical Corp, OH, USA) (11). While our TEE-based approach demonstrated only minimal esophageal stretch during deviation (approximately 3–4 mm), the application of suction allows the esophagus to be retracted around the device, thereby reducing its effective diameter. This mechanism likely accounts for the greater separation distances reported with suction-enabled systems.

In our cohort, successful deviation—defined as a safety margin >10 mm—was achieved in 78% of PV pairs, versus only 30% at baseline. This translated into a reduction of PV–esophagus overlap from more than half of PV pairs to only 2%.

These findings highlight the dynamic nature of esophageal positioning and demonstrate that mechanical displacement can substantially alter the anatomic determinants of esophageal risk. This observation is consistent with earlier non-controlled studies using TEE probes, endoscopes, or improvised stylets, which reported substantial esophageal mobility during AF ablation (6–10).

### Impact on esophageal thermal exposure

Beyond anatomic separation, a clinically relevant observation is the striking reduction in esophageal temperature elevation during radiofrequency ablation. In the deviation group, temperature rises >38 °C were rare, occurred in only two patients, and were limited to two radiofrequency applications overall. In contrast, nearly all patients in the standard care group experienced temperature elevations, with a median of three RF interruptions per patient.

Notably, when temperature increases did occur, the distance between the ablation catheter and the thermal probe was similar in both groups, and temperature rises were observed only at short catheter–probe distances (always <12 mm). This observation supports the relevance of the 10-mm safety distance threshold adopted in our study, as clinically relevant temperature elevations were only observed when the catheter–esophagus separation approached or fell below this range.

Although luminal esophageal temperature monitoring remains an imperfect surrogate for true esophageal wall injury (16, 18), the magnitude of reduction observed in the present study strongly suggests a clinically meaningful decrease in esophageal thermal exposure. Given that esophageal injury is believed to be a cumulative phenomenon related to repeated or sustained heating, the near elimination of temperature alerts likely represents a substantial safety gain.

### Safety considerations

TEE during cardiac procedures has been associated with a 0.67% risk of injuries (19), with complications ranging from minor oropharyngal bleeding to exceptional esophageal perforation (0.05%). A central concern with any mechanical deviation strategy is the potential for increased risk of esophageal trauma due to excessive stretching or focal pressure, and to repeated manipulations.

In the present study, we did not observe any acute or delayed clinical esophageal complications during follow-up, which is consistent with prior reports indicating that controlled mechanical deviation is generally well tolerated (9, 12, 13). This apparent favorable safety profile using TEE could be linked with the modest esophageal diameter increase during deviation (mean stretch of 3.5 mm) and with several technical aspects: avoidance of advancing the TEE probe while fixed in a curved position, gradual probe withdrawal to align maximal deviation with the target ablation zone, and continuous fluoroscopic monitoring.

Although systematic post-procedural endoscopy was not performed, the absence of symptoms and the marked reduction in thermal exposure together suggest a low likelihood of clinically relevant occult injury. However, larger studies including endoscopic evaluation will be necessary for confirming the safety profile and efficacy of this deviation technique.

### Potential implications in the PFA era

Despite the exponential growth of PFA, radiofrequency remains a widely used and extensively studied energy source for AF ablation, supported by three decades of clinical experience, continuous technological refinements, and broader versatility for ablation beyond pulmonary vein isolation. Moreover, the higher procedural costs associated with PFA may limit its widespread adoption in some healthcare systems.

From an esophageal safety perspective, however, the increasing use of PFA may substantially reduce the clinical relevance of strategies specifically designed to prevent thermal esophageal injury. Indeed, PFA has only been associated with acute non-transmural esophageal lesions that resolve by 14 days (20) without clinical implications (21), and no atrioesophageal fistula has been reported to date. In this context, routine esophageal temperature monitoring or mechanical esophageal deviation may be of limited value when PFA is used exclusively for PV isolation. Nevertheless, radiofrequency may remain relevant for additional ablation beyond PV isolation, including linear lesions and treatment of complex atrial substrates, and may also be required as a complementary energy source during PFA procedures. Therefore, mechanical esophageal deviation may retain a role in contemporary AF ablation, particularly in procedures involving radiofrequency applications on the posterior left atrium, either as the primary energy source or as an adjunct to PFA. In this setting, the ability of the TEE probe to deflect the esophagus may provide a simple and readily available means of reducing esophageal thermal exposure whenever radiofrequency energy is required.

### Study limitations

This study has several limitations. First, TEE imaging and probe manipulation requires proper training, which may not be part of routine skill set of all electrophysiologists.

Group allocation was operator-based rather than randomized, introducing potential selection and performance biases. Procedural strategies differed between operators, particularly for persistent AF. However, pulmonary vein isolation and posterior wall exposure—the principal determinants of esophageal risk—were comparable between groups, supporting the validity of the esophageal findings.

The sample size was limited and not powered to detect rare but catastrophic complications such as esophageal perforation or atrioesophageal fistula, whose incidence is typically well below 0.1% (22), and larger studies with systematic endoscopic follow-up would be necessary to establish the safety profile of this deviation technique.

Also, esophageal deviation was evaluated in this study in the setting of radiofrequency ablation. As discussed previously, PFA carries minimal esophageal risks and in procedures using exclusively PFA on left atrial posterior wall, esophageal protection techniques are likely irrelevant.

Importantly, esophageal injury assessment relied on clinical follow-up rather than systematic endoscopy. While this reflects contemporary practice and avoids unnecessary invasive testing, subclinical mechanical lesions may have been missed.

## Conclusion

Mechanical esophageal deviation using a standard TEE probe is a feasible and effective technique for increasing esophageal separation from posterior PV ablation sites and for markedly reducing esophageal thermal exposure during radiofrequency AF ablation. Despite the growing adoption of PFA, radiofrequency energy remains widely used, even more so for adjunctive lesions, linear ablation, and complex substrates. Esophageal protection therefore continues to be a critical issue in contemporary AF ablation practice. TEE-guided esophageal deviation offers a pragmatic, low-cost, and immediately implementable strategy to enhance procedural safety in centers without access to dedicated deviation devices and already performing TEE-guided transseptal punctures.

## Data Availability

The raw data supporting the conclusions of this article will be made available by the authors, without undue reservation.
